# Crimean-Congo Hemorrhagic Fever Virus for Clinicians—Epidemiology, Clinical Manifestations, and Prevention

**DOI:** 10.3201/eid3005.231647

**Published:** 2024-05

**Authors:** Maria G. Frank, Gretchen Weaver, Vanessa Raabe, State of the Clinical Science Working Group of the National Emerging Pathogens Training Education Center’s Special Pathogens Research Network

**Affiliations:** Denver Health and Hospital Authority, Denver, Colorado, USA (M.G. Frank);; University of Colorado School of Medicine, Denver (M.G. Frank);; University of Massachusetts Chan Medical School, Worchester, Massachusetts, USA (G. Weaver);; New York University Grossman School of Medicine, New York, New York, USA (V. Raabe)

**Keywords:** Crimean-Congo hemorrhagic fever, viruses, vector-borne infections, bunyavirus, viral hemorrhagic fever, countermeasure, vaccine, treatment

## Abstract

Crimean-Congo hemorrhagic fever (CCHF) is a tickborne infection that can range from asymptomatic to fatal and has been described in >30 countries. Early identification and isolation of patients with suspected or confirmed CCHF and the use of appropriate prevention and control measures are essential for preventing human-to-human transmission. Here, we provide an overview of the epidemiology, clinical features, and prevention and control of CCHF. CCHF poses a continued public health threat given its wide geographic distribution, potential to spread to new regions, propensity for genetic variability, and potential for severe and fatal illness, in addition to the limited medical countermeasures for prophylaxis and treatment. A high index of suspicion, comprehensive travel and epidemiologic history, and clinical evaluation are essential for prompt diagnosis. Infection control measures can be effective in reducing the risk for transmission but require correct and consistent application.

Human Crimean-Congo hemorrhagic fever (CCHF) infection mainly occurs after the bite of an infected tick or exposure to blood or tissues from infected animals; human-to-human transmission, particularly in healthcare settings, has also been reported. Approximately 10,000–15,000 cases of CCHF occur annually worldwide, although more definitive numbers are difficult to ascertain; up to 88% of cases are thought to be subclinical (*1*–*3*), unrecognized, or occur in locations with limited disease surveillance or laboratory testing capability (*4*,*5*). A recent meta-analysis of CCHF-endemic areas reported an overall acute infection prevalence of 22.5%, recent infection seroprevalence of 11.6%, and an overall past infection seroprevalence of 4.3% in humans (*6*).

CCHF causes clinical manifestations in humans ranging from asymptomatic infection to severe hemorrhagic fever. The case-fatality rate (CFR) during outbreaks is typically 5%–30% (*1*), but CFRs of up to 62% have been reported (*7*). Disease caused by CCHF virus (CCHFV) is limited to humans, but asymptomatic transient viremia (lasting <15 days) has been documented in livestock and wild animals (*8*). Severe or fatal disease causes proinflammatory immune response that leads to vascular dysfunction, disseminated intravascular coagulation, multiorgan failure, and shock (*9*). The detection of IgM (present as early as day 4–5 of illness) and IgG (present after days 7–9 of illness) correlates with declining viremia, but fatal cases often show no or very late immune response (*10*). However, antibody response to CCHFV does not correlate with disease outcome or protection from vaccines, which, combined with a paucity of available animal models (*11*), makes research on vaccines and treatments challenging. No vaccines or treatments for CCHF have been approved by the US Food and Drug Administration.

This second article in a 3-part series summarizing the main aspects of CCHF is intended to provide clinicians with an overview of the epidemiology, clinical features, and prevention and control of CCHF. The first article focuses on the virology, pathogenesis, and pathology of CCHF (*12*) and the third on diagnostic testing and management of CCHF (*13*).

## Methods

The focused review for this paper involved MeSH (National Center for Biotechnology [NCBI], https://www.ncbi.nlm.nih.gov/mesh) and PubMed (https://www.pubmed.ncbi.nlm.nih.gov)search strings customized for CCHF and CCHFV. We focused our review on the past 10 years and used human data when available; we included older relevant data and animal data where appropriate. We conducted title, abstract, and full text reviews of relevant manuscripts, reviews, and book chapters. We also completed bibliography scans on review articles and meta-analyses.

## Epidemiology

**Figure 1:**
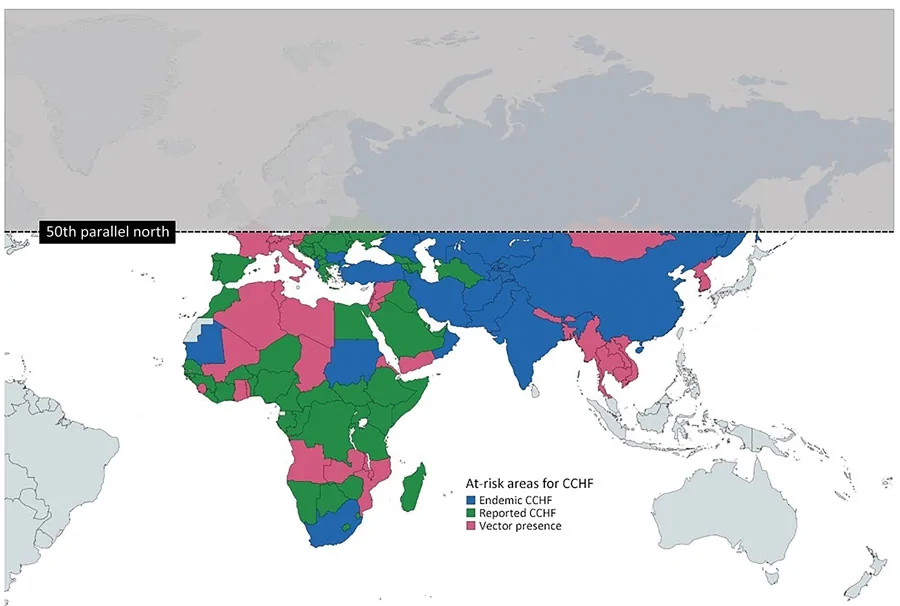
Geographic distribution of CCHF and *Hyalomma* spp. ticks. CCHF, Crimean-Congo hemorrhagic fever.

CCHF is the most geographically widespread tickborne disease, identified in >30 countries in Africa, Asia, the Middle East, and Europe located south of the 50th parallel north (Figure 1). The annual incidence is estimated to be 10,000–15,000 cases worldwide but has been slowly and steadily rising (*3*). That increase in incidence is thought to be caused by the expanding range of its main vector, *Hyalomma* ticks, and by increased testing (*6*). Most cases occur after tick bites; the second most common means of exposure is through bodily fluids and tissue from infected animals; and last, human-to-human transmission can occur in the healthcare setting.

In recent years, CCHF has been documented in previously unaffected countries, such as Spain and Jordan (*14*–*17*). Although tickborne transmission is the main route for human CCHF, contact with viremic animals, infected humans, or contaminated surfaces (e.g., nosocomial transmission) can also lead to human illness. Persons at the highest risk for CCHF include farmers living in CCHF-endemic areas, participants in recreational activities (e.g., hiking, camping) in endemic areas, slaughterhouse workers, veterinarians, and healthcare workers, who are now considered the second most affected group (*3*,*18*). Transmission to household contacts is uncommon, although horizontal transmission from mother to child has been reported (*19*). Sexual transmission has been proposed; however, presence of CCHFV in semen or vaginal fluids has yet to be confirmed (*20*). Similarly, airborne transmission has been hypothesized to occur in association with nosocomial and laboratory-acquired CCHF clusters, despite a lack of direct evidence (*21*–*23*). Nosocomial infections are symptomatic in 92.4% of cases, and in 76.5% of those patients, hemorrhagic disease develops; these cases tend to have high mortality (CFR 32.4%) (*23*).

## Clinical Features

### Incubation Period 

The typical incubation period for CCHF is 3–7 days (range 1–13 days); incubation period is shorter (1–5 days) after a tick bite and longer (5–13 days) after exposure to infected blood or tissues (*14*). The accelerated viral dissemination after a tick bite is thought to be caused by a tick saliva–enabling effect, known as saliva-activated transmission, related to bioactive molecules in tick saliva causing antihemostatic, antiinflammatory, and immunomodulatory effects on the vertebrate host (*14*,*24*).

### Clinical Spectrum of Infection 

**Figure 2:**
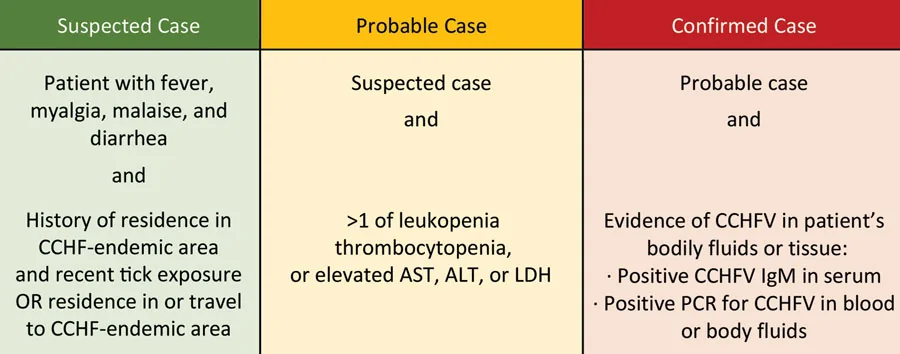
Crimean-Congo hemorrhagic fever case definitions, modified from Ergonul et al. (*1*). ALT, alanine aminotransferase; AST, aspartate aminotransferase; CCHF, Crimean-Congo hemorrhagic fever; CCHFV, CCHF virus; LDH, lactate dehydrogenase.

Clinical manifestations of CCHF range from asymptomatic (<88%) (*3*) infection or mild, nonspecific febrile illness to severe hemorrhagic disease with multiorgan failure leading to death (*14*). CCHF case definitions vary across endemic regions; the case definition proposed in Ergonul et al. (*1*) includes suspect, probable, and confirmed cases (Figure 2).

### Clinical Course 

**Figure 3:**
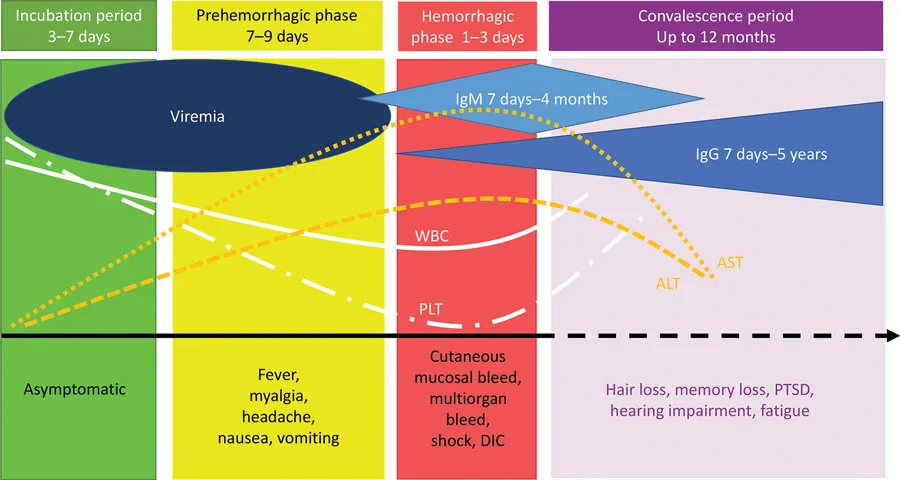
Classic clinical disease course of Crimean-Congo hemorrhagic fever. ALT, alanine aminotransferase; AST, aspartate aminotransferase; DIC, disseminated intravascular coagulation; PLT, platelet count; PTSD, posttraumatic stress disorder; WBC, white blood cell count.

CCHF is characterized by an incubation period, as described, followed by prehemorrhagic, hemorrhagic, and convalescent phases (Table; Figure 3). Most patients will recover and transition to the convalescent period; patients who die typically succumb to the disease by day 10.

**Table:** Clinical phases of Crimean-Congo hemorrhagic fever*

Clinical phase	Duration	Clinical features	Laboratory features
Incubation	3–7 d (3–5 d after tick bite, 5–7 d after exposure to blood or tissue)	Not applicable	Normal-mildly decreased PLT
Prehemorrhagic	1–7 d	Fever, headache, myalgia, dizziness, nausea, vomiting, diarrhea, hyperemia of upper body, conjunctivitis	Viremia (positive PCR), mild leukopenia, mild thrombocytopenia, elevated CK, mild elevation of AST, ALT, and LDH
Hemorrhagic	Begins at day 3–5 of illness	Petechial rash (skin, conjunctiva, mucosa), large cutaneous ecchymoses, gastrointestinal and genitourinary bleeding, hepatosplenomegaly, if fatal (days 5–14 of illness) secondary to MOF, bleeding, shock DIC	Decreasing viremia, in most cases resolved by day 9 of illness, positive serum IgM against CCHFV, leukopenia, anemia, profound thrombocytopenia, marked elevation of AST, elevation of ALT, elevated PT, aPTT, D-dimer and FDP, schistocytes
Convalescence	Up to 1 y	Weakness, malaise, hair loss, anorexia, polyneuritis, impaired memory, vision impairment, hepatic and renal insufficiency	Thrombocytosis, slow decrease in AST and ALT, slow resolution of renal and liver function, positive serum IgG against CCHFV

*ALT, alanine aminotransferase; aPTT, activated partial thromboplastin time; AST, aspartate aminotransferase; CCHFV, Crimean-Congo hemorrhagic fever virus; CK, creatine phosphokinase; DIC, disseminated intravascular coagulation; FDP, fibrinogen degradation products; LDH, lactate dehydrogenase; MOF, multiorgan failure; PT, prothrombin time.

The prehemorrhagic phase frequently lasts 1–5 days and is usually characterized by nonspecific symptoms. Those symptoms include sudden onset of fever, which lasts for an average of 4–5 days, and nonspecific signs and symptoms such as diarrhea, dizziness, headache, myalgia, nausea, vomiting, and weakness. Headache occurs in almost 70% of patients and tends to be severe. Two thirds of patients describe the pain as mimicking a migraine crisis, including throbbing and being accompanied by nausea, vomiting, photophobia, and phonophobia (*25*); half of patients describe the headache as worsening with activity. Characteristically, those patients might also develop upper body (face, neck, and chest) hyperemia, conjunctivitis, and congested sclera. Because of the lack of specificity in clinical manifestations, a high index of suspicion on the basis of a thorough exposure and travel history is essential for recognition.

The hemorrhagic illness phase typically begins 3–5 days after symptom onset and is usually short, lasting 1–3 days. This phase begins with a petechial rash of the skin and mucous membranes and might progress to more severe hemorrhagic features at multiple sites, including ecchymoses; cerebral hemorrhage; bleeding from the nasopharynx, gastrointestinal tract (hematemesis and melena), and genitourinary (hematuria) tract; menometrorrhagia; and hemoptysis (*14*). Epistaxis is present in <50% of patients in the hemorrhagic phase, hematemesis in <35% of patients, hematuria, melena and hematochezia in 10%–20% of patients, and intraabdominal or intracerebral bleeding in 1%–2% of cases (*1*). Large ecchymoses are present in 30%–45% of patients, and although they are not pathognomonic, their presence should suggest CCHF over other viral hemorrhagic fevers. Hepatosplenomegaly is common and described in up to one third of patients (*1*). Severe disease during this phase is often characterized by anemia, thrombocytopenia, evidence of coagulation abnormalities (prolonged prothrombin time [PT] and activated partial thromboplastin time [aPTT]) and disseminated intravascular coagulation. Liver enzymes, including alanine aminotransferase (ALT) and aspartate aminotransferase (AST), are typically elevated. Renal insufficiency and hypotension are common in severe cases (*14*,*26*,*27*).

During the hemorrhagic phase, patients might experience neurologic and neuropsychiatric symptoms such as agitation, confusion, delusions, neck stiffness, headache, photophobia, and, in rare cases (2.8%), myoclonic jerks (*28*). Involvement of the central nervous system has been suspected; however, a recent prospective study showed no cases with encephalitis or brain abnormalities on magnetic resonance imaging despite a high percentage of patients experiencing fever (94.4%) and headache (66.7%). None of the 36 patients in the case series showed brain changes over the course of their disease, although no cerebrospinal fluid analysis was performed in the study, so presence of viral meningitis could not be ruled out (*28*,*29*). Those findings in humans are in contrast with a study of humanized mice infected with CCHFV in which autopsies showed gliosis, meningitis, and meningoencephalitis, suggesting direct viral infection of the central nervous system (*11*,*17*,*30*).

Cardiopulmonary manifestations include myocardial infarction, myocarditis (*31*), pulmonary edema, and pleural effusions. Engin et al. (*32*) evaluated 44 consecutive CCHF patients using transthoracic echocardiography and reported that patients with severe CCHF had statistically (but not necessarily clinically) significant lower ejection fraction of the left ventricle (50% vs. 55%) and higher systolic pulmonary pressures and were more likely to have pericardial effusion than were nonsevere CCHF patients. Whether myocardial dysfunction is a result of immune-related or direct viral cytotoxic effect on the myocardium is unclear.

Literature case reports of CCHF-associated acute pancreatitis and acute nonsuppurative parotitis during the hemorrhagic phase of illness can be found, but no virologic confirmation in tissue was obtained in those cases (*33*,*34*). A case of acute epididymo-orchitis during the prehemorrhagic phase has also been reported (*35*). Most deaths occur in the second week of illness and are associated with rapidly developing refractory shock that leads to multiorgan failure and severe coagulopathy with evidence of acute and severe hepatopathy (*14*,*36*,*37*).

The convalescent phase of CCHF usually starts on day 10–20 of illness and can last up to 1 year. Most patients recover without complications or sequelae. Among those patients with symptomatic convalescence, they frequently experience fatigue and malaise, hair loss, anorexia, and polyneuritis. Tachycardia and dyspnea have also been described. Memory and visual and auditory impairment have also been described (*1*,*21*). A study from Turkey reported that 48.4% of patients studied exhibited symptoms of posttraumatic stress disorder (PTSD) and 18.5% had PTSD after recovery (*38*). PTSD and PTSD symptoms were more common among patients who had required intensive care unit stays (*38*).

To date, relapses of CCHF and reinfections with CCHFV, particularly of patients being reexposed in endemic areas, have not been described (*10*,*14*,*39*). Nonetheless, duration of protective immunity has not yet been elucidated.

### Special Populations 

More than 40 cases of CCHF in pregnant women have been reported and are associated with high maternal mortality (CFR 34%) compared with nonpregnant patients (CFR 4%–14% depending on the reporting country); mortality rates were higher in the second half of pregnancy, but the difference was not statistically significant. Fetal and neonatal mortality (58%) is associated with spontaneous abortion or maternal death. Exposure to bodily fluids (i.e., blood, amniotic fluid) during cesarean section or vaginal delivery confers a high risk for transmission; up to 14.8% of deliveries have resulted in transmission to healthcare workers (*40*). It is key to consider HELLP (hemolysis, elevated liver enzymes, low platelets) syndrome in the differential diagnosis of pregnant patients suspected to have CCHF (*1*).

Most pediatric cases of CCHF are the result of a tick bite, and patients more frequently exhibited rash, abdominal pain, and myalgia, leading to a different differential diagnosis than seen in adults. Tonsillopharyngitis is a common finding in pediatric patients (*41*). Elevated AST, ALT, and lactate dehydrogenase, as well as leukopenia and thrombocytopenia, are common among pediatric patients admitted for CCHF management (*1*). A case of acute CCHF-related myocarditis in a 13-year-old was reported; symptoms resolved completely after the convalescent period (*31*). The clinical course for reported pediatric cases was milder and shorter than for adults (*41*).

Pediatric cases of hemophagocytic lymphohistiocytosis (HLH) secondary to CCHF have been described (*42*,*43*). Secondary HLH, although rare, can be associated with malignancies, severe infections, medications, and autoimmune disorders and has been thought to be secondary to a hyperinflammatory syndrome (*44*). Most patients will have a combination of fever, hepatosplenomegaly, pancytopenia, hypertriglyceridemia, hypofibrinogenemia, a variety of neurologic symptoms, and evidence of hemophagocytosis in pathology examination of bone marrow or other tissues (*44*). Because of the high rates of illness and death associated with HLH, its early recognition is key for timely treatment consideration (such as corticosteroids, intravenous immunoglobulin, immunomodulators, and therapeutic plasma exchange) (*43*,*44*).

### Disease Severity and Mortality Risk Factors 

CFR estimates range from 5% to 60% in case series depending on geographic region. Multiple factors, such as healthcare resource availability, difference in circulating strain virulence, risk for co-infections, and the clinician’s threshold for early CCHF testing, can affect outcomes (*14*,*37*,*45*).

Several CCHF disease severity assessment models have been proposed. In 1989, Swanepoel et al. (*36*) proposed a model that predicted a >90% fatal outcome if patients had any of the following: leukocytosis (leukocytes >10,000/mm^3^), thrombocytopenia (platelets <20,000/mm^3^), AST >200 U/L or ALT >150 U/L, aPTT >60 seconds, or fibrinogen <110 mg/dL. In 2006, Ergonul et al. (*46*) defined severe CCHF as the presence of any of thrombocytopenia (<20,000/mm^3^), AST >700 U/L or ALT >900 U/L, aPTT >60 seconds, or fibrinogen <110 mg/dL, in addition to the presence of melena, hematemesis, or somnolence. In both models, criteria were based on signs and symptoms that appeared within 5 days after symptom onset (*36*,*47*). 

Bakir et al. (*47*) developed a scoring system for CCHF severity to aid in predicting clinical course and mortality risk through a severity grading score (SGS). The variables used in the SGS system are age, routinely collected and available laboratory markers (PT, aPTT, international normalized ratio [INR], AST, ALT, lactate dehydrogenase, and leukocyte and platelet counts), and other clinical features (hepatomegaly, organ failure, bleeding), each with associated point values. Point values predicted mortality risk (low, SGS <4; medium, SGS 5–8; high, SGS >9): patients with a high SGS at admission were at high risk for death (sensitivity 96%, specificity 100%), whereas a low score showed no association with mortality; mortality risk was 20% in the medium risk group (*47*).

In 2022, Bakir et al. (*37*) published a comparison of models’ performance in predicting death in CCHF patients. The authors compared the sequential organ failure assessment score, the qSOFA (quick sepsis-related organ failure assessment), APACHE II score, and SGS. All models except qSOFA were adequate for predicting death when applied at admission; however, all models performed well at 72 hours and 120 hours after admission (*37*).

CCHF viremia levels have been correlated with disease severity, and viral loads equal or above 10^8^ copies/mL and 10^9^ copies/mL are significantly associated with high mortality from CCHF (*48*,*49*). However, CCHF viral load measurements are not routinely available to the bedside clinician.

### Differential Diagnoses 

The differential diagnosis for CCHFV infection might vary geographically and is based on known occupation and environmental exposures, immunization status, season, and the geographic location (current and recent) of the patient. Options include, but are not limited to, brucellosis, COVID-19, ehrlichiosis, influenza, leptospirosis, Lyme disease, malaria, Q fever, rickettsiosis, salmonellosis, tickborne encephalitis, viral hepatitis, and other viral hemorrhagic fevers (*1*). Obtaining a thorough history, including animal, environmental, insect, occupational, and travel exposures, is critical for assessing the likelihood of CCHF as a potential diagnosis.

## Infection Prevention and Control

Infection prevention and control measures against CCHF aim to minimize exposure. Such measures apply to community, occupational, and healthcare settings.

### Community Settings

The risk of acquiring CCHF in the community is primarily related to exposure to ticks or infected animals. Thus, prevention efforts focus on prevention of tick-to-human transmission (e.g., wearing protective clothing, avoiding locations with high tick burden) and animal-to-human transmission (e.g., use of gloves and other protective clothing for direct contact with animals’ bodily fluids and their tissues in CCHF-endemic areas) (*3*,*18*).

CCHFV does not typically cause disease in animals, although tick infestation of domestic, farm, and wild animals can increase the risk for transmission to humans. Reducing activities in tick-infested areas and implementing pest-management strategies in both domestic and farm animals are key for preventing CCHF transmission in agricultural communities (*18*). Other proposed community strategies to mitigate the effects of CCHF include regulating and monitoring livestock migratory activities, media campaigns focusing on simple CCHF prevention measures and community engagement, easy-to-access training modules for healthcare workers, and increased communication between veterinarian and medical health experts (*3*,*50*).

Temporal trends in incidence could help guide the timing of community mitigation efforts for maximum impact. CCHF follows a seasonal pattern and is positively associated with monthly average temperature, monthly cumulative rainfall, and decreased relative humidity (Appendix reference *51*). In addition, increases in CCHF cases often occur during or around the time of the annual celebration of Eid al‐Adha. Rural livestock brought to urban areas for slaughter for the festivities might carry CCHFV (either through infected ticks or because livestock are viremic at the time of slaughter) (*50*). Geographic areas where risk for CCHF is higher can be targeted for control strategies using a predictive tool to estimate the prospective number of CCHF cases for the next 2 years (*3*,*50*).

### Occupational Settings (Nonhealthcare)

Persons whose occupations expose them to animals or raw animal tissues and fluids, such as butchers, farmers, slaughterhouse workers, veterinarians, and veterinary clinic staff, are at increased risk for CCHF exposure (*3*; Appendix reference *52*). Availability and use of PPE when handling animals, animal carcasses, or animal body fluids, as well as the quarantining of livestock potentially carrying CCHFV or CCHFV-infected ticks before transport and slaughter, can also minimize human exposure in those occupations (*18*).

### Healthcare Settings

Education on identifying signs and symptoms of CCHF early, rapidly isolating suspect cases, and informing the appropriate authorities, as well as on obtaining information on relevant epidemiologic history or exposures, is essential to reducing risk for nosocomial transmission. Human-to-human transmission is most often documented in the nosocomial setting and is thought to occur through exposure to blood and bodily fluids of infected patients. Numerous case series have described clusters of CCHF among healthcare workers in Pakistan, Russia, Turkey, Mauritania, Iran, and elsewhere; failures in infection prevention and control have been implicated (*22*; Appendix references *53–57*). In 1 study, the seroprevalence of healthcare workers who cared for CCHF patients was 3.78%, compared with 0% for healthcare workers with no known exposure to CCHF (Appendix reference *55*). A delay in clinical suspicion of CCHF and subsequent delay in implementing infection control measures has also been reported as a contributing factor in nosocomial transmission (Appendix reference *58*).

Persons who are suspected of having CCHF should be isolated immediately to minimize the risk for nosocomial transmission, appropriate PPE should be used when providing care, and relevant public health authorities should be informed (*3*). Healthcare worker PPE for the management of CCHF patients is generally based on recommendations for other viral hemorrhagic fevers, mainly filoviruses, such as Ebola virus disease. Both the World Health Organization (https://www.who.int/health-topics/crimean-congo-haemorrhagic-fever) and the US Centers for Disease Control and Prevention (https://www.cdc.gov/vhf/crimean-congo/index.html) apply infection control approaches for Ebola virus disease to management of patients with suspected or confirmed CCHF. That guidance includes detailed recommendations on placing and isolating patients, collecting and processing laboratory specimens, managing waste, and cleaning and disinfecting the environment (https://www.cdc.gov/vhf/ebola/clinicians/evd/infection-control.html).

Needlestick injuries and splash exposures to mucous membranes are considered common mechanisms of exposure for nosocomial CCHFV transmission from blood. Other body fluids might potentially transmit CCHFV; CCHFV RNA can be detected in saliva and urine early in the clinical course. Further research regarding timing of viral presence in other bodily fluids is necessary (Appendix references *59,60*). Policies and procedures for isolation, discharge criteria, and guidance on the potential risk for transmission after discharge should take into account the potential for persistent viral shedding (*20*; Appendix references *59–61*). The patient should be placed in a single room, when available, immediately upon suspicion of CCHF. Although airborne transmission has been proposed in some nosocomial clusters, definitive evidence is lacking to recommend universal use of N95 respirators for the care of CCHF patients; however, N95 respirators or equivalent should be worn during aerosol-generating procedures (*22*). As has been noted for other viral hemorrhagic fever diseases, the patient’s severity of illness seems to correlate with increased risk for infections in healthcare workers (Appendix reference *61*). Despite availability of infection prevention and control guidelines, a recent survey of 23 international centers taking care of CCHF patients in endemic countries noted high variability in healthcare workers’ use of PPE; all centers reported a high-risk exposure in the previous 5 years (Appendix references *61,62*).

## Conclusion

CCHF is the most geographically widespread tickborne disease, identified in >30 countries in Africa, Asia, the Middle East, and Europe located south of the 50th parallel north. It poses a continued public health threat; estimated annual incidence is 10,000–15,000 cases worldwide. Farmers, persons participating in outdoor recreational activities, slaughterhouse workers, veterinarians, and healthcare workers in CCHF-endemic areas are at risk for infection. Clinical manifestations of CCHF range from asymptomatic infection or mild, nonspecific febrile illness to severe hemorrhagic disease with multiorgan failure ultimately leading to death; reported CFR in some case series is as high as 60% (*7*). A high index of suspicion, comprehensive travel and epidemiologic history, and clinical evaluation are essential for prompt diagnosis. Infection control measures can be effective in reducing the risk for transmission both within community and healthcare settings; however, correct and consistent application is required to effectively achieve this goal.

Appendix Additional information about Crimean-Congo hemorrhagic fever virus for clinicians—epidemiology, clinical manifestations, and prevention
