# Supplementary material for: Crimean-Congo Hemorrhagic Fever Virus for Clinicians—Epidemiology, Clinical Manifestations, and Prevention
Source: Emerg Infect Dis. 2024 May;30(5):854–63. doi: 10.3201/eid3005.231647 (PMC11060446; doi:10.3201/eid3005.231647)
Supplement: Appendix — Additional information about Crimean-Congo hemorrhagic fever virus for clinicians—epidemiology, clinical manifestations, and prevention [file 23-1647-Techapp-s1.pdf]

Article DOI: https://doi.org/10.3201/eid3005.231647

EID cannot ensure accessibility for supplementary materials supplied by authors. Readers who have difficulty accessing supplementary content should contact the authors for assistance.

Crimean-Congo Hemorrhagic Fever Virus for Clinicians—Epidemiology, Clinical Manifestations, and Prevention

Appendix

Additional References

51. Nili S, Khanjani N, Jahani Y, Bakhtiari B. The effect of climate variables on the incidence of Crimean Congo hemorrhagic fever (CCHF) in Zahedan, Iran. BMC Public Health. 2020;20:1893. PubMed https://doi.org/10.1186/s12889-020-09989-4

52. Mourya DT, Yadav PD, Gurav YK, Pardeshi PG, Shete AM, Jain R, et al. Crimean Congo hemorrhagic fever serosurvey in humans for identifying high-risk populations and high-risk areas in the endemic state of Gujarat, India. BMC Infect Dis. 2019;19:104. PubMed https://doi.org/10.1186/s12879-019-3740-x

53. Burney MI, Ghafoor A, Saleen M, Webb PA, Casals J. Nosocomial outbreak of viral hemorrhagic fever caused by Crimean hemorrhagic fever-Congo virus in Pakistan, January 1976. Am J Trop Med Hyg. 1980;29:941–7. PubMed https://doi.org/10.4269/ajtmh.1980.29.941

54. Leblebicioglu H, Sunbul M, Guner R, et al. Healthcare-associated Crimean-Congo haemorrhagic fever in Turkey, 2002–2014: a multicentre retrospective cross-sectional study. Clin Microbiol Infect. 2016;22:387.e1–4. **PMID 26806137**

55. Mardani M, Rahnavardi M, Rajaeinejad M, Naini KH, Chinikar S, Pourmalek F, et al. Crimean-Congo hemorrhagic fever among health care workers in Iran: a seroprevalence study in two endemic regions. Am J Trop Med Hyg. 2007;76:443–5. PubMed https://doi.org/10.4269/ajtmh.2007.76.443

56. Nabeth P, Cheikh DO, Lo B, Faye O, Vall IO, Niang M, et al. Crimean-Congo hemorrhagic fever, Mauritania. Emerg Infect Dis. 2004;10:2143–9. PubMed https://doi.org/10.3201/eid1012.040535

57. Schuster I, Mertens M, Köllner B, Korytář T, Keller M, Hammerschmidt B, et al. A competitive ELISA for species-independent detection of Crimean-Congo hemorrhagic fever virus specific antibodies. Antiviral Res. 2016;134:161–6. PubMed https://doi.org/10.1016/j.antiviral.2016.09.004

58. Conger NG, Paolino KM, Osborn EC, Rusnak JM, Günther S, Pool J, et al. Health care response to CCHF in US soldier and nosocomial transmission to health care providers, Germany, 2009. Emerg Infect Dis. 2015;21:23–31. PubMed https://doi.org/10.3201/eid2101.141413

59. Leblebicioglu H, Sunbul M, Bodur H, Ozaras R, Barut S, Buyuktuna SA, et al.; Crimean–Congo Haemorrhagic Fever Research Network of Turkey (CCRNT). Discharge criteria for Crimean-Congo hemorrhagic fever in endemic areas. J Infect. 2016;72:500–1. PubMed https://doi.org/10.1016/j.jinf.2016.01.009

60. Leblebicioglu H, Sunbul M, Barut S, Buyuktuna SA, Ozkurt Z, Yapar D, et al.; Crimean Congo Hemorrhagic Fever Research Network of Turkey. Multi-center prospective evaluation of discharge criteria for hospitalized patients with Crimean-Congo hemorrhagic fever. Antiviral Res. 2016;133:9–13. PubMed https://doi.org/10.1016/j.antiviral.2016.07.010

61. Fletcher TE, Gulzhan A, Ahmeti S, Al-Abri SS, Asik Z, Atilla A, et al. Infection prevention and control practice for Crimean-Congo hemorrhagic fever—a multi-center cross-sectional survey in Eurasia. PLoS One. 2017;12:e0182315. PubMed https://doi.org/10.1371/journal.pone.0182315

62. World Health Organization. Interim infection prevention and control guidance for care of patients with suspected or confirmed filovirus haemorrhagic fever in health-care settings, with focus on Ebola [cited 2023 Nov 12]. https://apps.who.int/iris/handle/10665/1305962014:https://apps.who.int/iris/handle/10665/130596
